# Mitotic defects in fission yeast lipid metabolism ‘cut’ mutants are suppressed by ammonium chloride

**DOI:** 10.1093/femsyr/foy064

**Published:** 2018-06-19

**Authors:** Róbert Zach, Jarmila Tvarůžková, Martin Schätz, Ondřej Ťupa, Beáta Grallert, Martin Převorovský

**Affiliations:** 1 Department of Cell Biology, Faculty of Science, Charles University, Prague, Czech Republic; 2 Department of Computing and Control Engineering, University of Chemistry and Technology, Prague, Czech Republic; 3 Department of Radiation Biology, Institute for Cancer Research, Oslo University Hospital, Oslo, Norway

**Keywords:** *Schizosaccharomyces pombe*, *cut6*, *cbf11*, mitosis, ammonium chloride, cell cycle progression

## Abstract

Fission yeast ‘cut’ mutants show defects in temporal coordination of nuclear division with cytokinesis, resulting in aberrant mitosis and lethality. Among other causes, the ‘cut’ phenotype can be triggered by genetic or chemical perturbation of lipid metabolism, supposedly resulting in shortage of membrane phospholipids and insufficient nuclear envelope expansion during anaphase. Interestingly, penetrance of the ‘cut’ phenotype in mutants of the transcription factor *cbf11* and acetyl-coenzyme A carboxylase *cut6*, both related to lipid metabolism, is highly dependent on growth media, although the specific nutrient(s) affecting ‘cut’ occurrence is not known. In this study, we set out to identify the growth media component(s) responsible for ‘cut’ phenotype suppression in *Δcbf11* and *cut6–621* cells. We show that mitotic defects occur rapidly in *Δcbf11* cells upon shift from the minimal EMM medium (‘cut’ suppressing) to the complex YES medium (‘cut’ promoting). By growing cells in YES medium supplemented with individual EMM components, we identified ammonium chloride, an efficiently utilized nitrogen source, as a specific and potent suppressor of the ‘cut’ phenotype in both *Δcbf11* and *cut6–621*. Furthermore, we found that ammonium chloride boosts lipid droplet formation in wild-type cells. Our findings suggest a possible involvement of nutrient-responsive signaling in ‘cut’ suppression.

## Teaser

Ammonium chloride, a nitrogen source used in fission yeast growth media, was found to rescue cell division defects in mutants with perturbed lipid metabolism.

## INTRODUCTION

Faithful progression through cell cycle phases is essential for successful cell reproduction and transmission of genetic information to daughter cells. The whole process is tightly regulated and culminates with mitosis followed by cytokinesis. In standard growth media, the fission yeast *Schizosaccharomyces pombe* features very short G1 and S phases, a long G2 phase (∼70% of the cycle) and a rapid mitosis, during which the nuclear envelope does not break down. Thus, when grown in standard complex medium (YES; yeast extract with supplements), the daughter nuclei have already completed S phase by the time cytokinesis has finished (Sabatinos and Forsburg 2010). Interestingly, this timing can be influenced by manipulating G1 duration by providing the cells with different sources of nitrogen (Carlson *et al.*^1999^).

Numerous *S. pombe* mutants have been identified in which septation and/or cytokinesis erroneously take place in the absence of normal sister chromatid separation. This often results in the so-called ‘cut’ terminal phenotype of undivided nucleus being intersected by the septum (Uemura and Yanagida 1984; Hirano *et al.*^1986^; Samejima *et al.*^1993^; Saitoh *et al.*^1996^; Převorovský *et al.*^2009^). Most known ‘cut’ genes are directly involved in chromosome condensation, sister chromatid separation or anaphase progression (Yanagida 1998). Intriguingly, the ‘cut’ phenotype has also been described in mutants of several lipid metabolism genes (Saitoh *et al.*^1996^; Převorovský *et al.*^2009^) and chemical inhibition of lipid synthesis leads to the ‘cut’ phenotype (Saitoh *et al.*^1996^; Takemoto *et al.*^2016^). During anaphase in *S. pombe*, the nuclear envelope undergoes rapid expansion, and it has recently been shown that fatty acid synthesis and phospholipid production are critical for successful separation of daughter nuclei and proper chromosome segregation (Makarova *et al.*^2016^; Takemoto *et al.*^2016^).

Cbf11 is a transcription factor belonging to the CSL (CBF1/RBP-J_Κ_/Suppressor of Hairless/LAG-1) family (Převorovský, Půta and Folk 2007). Cbf11 regulates cell cycle progression and cell adhesion, and cells lacking *cbf11* show high incidence of the ‘cut’ phenotype when grown in YES (Převorovský et al. 2009, 2016; Kwon *et al.*^2012^). We have recently shown that Cbf11 regulates several lipid metabolism genes, including the essential *cut6* acetyl-coenzyme A carboxylase gene (Převorovský et al. 2015, 2016). Cut6 is the rate-limiting enzyme of fatty acid synthesis and the *cut6–621* mutant exerts the ‘cut’ phenotype at restrictive temperature. The precise nature of the *cut6–621* mutation is not known (Saitoh *et al.*^1996^). We have shown that decreased Cut6 activity likely contributes to the ‘cut’ phenotype of *Δcbf11* cells (Převorovský *et al.*^2016^). Curiously, several *Δcbf11*-associated defects can be suppressed by deletion of *pka1* or *sty1*, encoding the nutrient-sensing protein kinase A (PKA) catalytic subunit and general stress-response MAP kinase, respectively. Furthermore, the ‘cut’ phenotype of *Δcbf11* and *cut6–621* cells is largely diminished when cells are grown in the minimal defined EMM medium (Převorovský et al. 2015, 2016).

Temperature-sensitive mutations in *cut4* and *cut9*, encoding essential anaphase promoting complex (APC/C) subunits, cause loss of viability at increased temperature when cells are grown in complex, yeast extract-based YPD medium. Notably, the lethality can be suppressed by inactivating the PKA pathway in the *cut4–533* and *cut9–665* mutants, or by growing the cells in EMM medium in the case of *cut4–533* (Yamashita *et al.*^1996^; Yamada, Kumada and Yanagida 1997).

These observations collectively suggest that the availability of specific nutrients and/or signaling through nutrient-responsive pathways play an important role in the proper coordination of nuclear and cellular division. However, the nature of the nutrient(s) affecting this coordination is currently unknown. In this study, we set out to identify the EMM medium component(s) responsible for ‘cut’ phenotype suppression in the *Δcbf11* and *cut6–621* lipid metabolism mutants.

## MATERIALS AND METHODS

### Strains, media and cultivations

*Schizosaccharomyces pombe* strains used in this study were JB32 (*h^+^*), MP44 (*h^+^ Δcbf11::kanR*) (Převorovský *et al.*^2015^), MP218 (*h^+^ cut6–621*) (Saitoh *et al.*^1996^) and MP594 (*h^+^ ptl2:GFP-kanR Δcbf11::natR*) (Yang *et al.*^2016^). Unless indicated otherwise in the text, *S. pombe* cells were grown at 32°C according to standard procedures (Moreno, Klar and Nurse 1991). Temperature-sensitive strains were grown at 25°C, or at the semi-permissive temperature of 30°C. Cultivation media used in this study included the minimal defined EMM (Formedium, UK), complex YES (0.5% yeast extract, 3% glucose, 50 mg L^−1^ each of adenine, uracil, L-histidine, L-leucine and L-lysine) and YES variants supplemented with EMM-contained chemical compounds at concentrations listed in Table S1 (Supporting Information) (EMM composition as declared by the manufacturer). For medium shift experiments, exponentially growing *S. pombe* cells cultured in EMM were collected by centrifugation (1000 × g, 3 min, 25°C), resuspended in the same volume of fresh YES and incubated at 32°C. In all other experiments, cultures were grown in the indicated media for the whole duration of the experiment.

For growth rate measurements, *S. pombe* cells were first grown exponentially in YES. Culture volumes corresponding to ∼1.2 × 10^6^ cells were collected and centrifuged (1000 × g, 3 min, 25°C). Supernatants were removed and cell pellets were washed with the appropriate media. The resulting cell suspensions were then centrifuged again (1000 × g, 3 min, 25°C), supernatants were discarded, and cell pellets were resuspended in 1.5 mL of appropriate media. Aliquots of 1.4 mL of resulting cell suspensions were loaded into 12-well plates and introduced into the VarioSkan Flash plate reader (Thermo Scientific). Plates were incubated at 32°C with background shaking (180 spm, rotation diameter 20 mm). Optical densities were measured at 10 min intervals at λ = 595 nm. Doubling times (DT) were calculated according to the formula DT = 1/k, where k represents the slope of logarithmic phase of growth. Microsoft Excel 2007 was used for data processing and determination of k-value.

### Microscopy

For nuclear staining, exponentially growing *S. pombe* cells were collected by centrifugation (1000 × g, 3 min, 25°C) and fixed by resuspending in 70% ethanol. Ethanol-fixed cells were centrifuged again (1000 × g, 3 min, 25°C) and resuspended in deionized H_2_O. Cells were stained in suspension with 1 μg mL^−1^ 4^΄^,6-diamidine-2^΄^-phenylindole dihydrochloride (DAPI). Cell images were taken using the Olympus Cell R and Leica AF 6000LX microscopic systems. Frequency of ‘cut’ phenotype occurrence was determined by manual counting of ‘cut’ cells using the ImageJ software, version 1.51j8 (Schneider, Rasband and Eliceiri 2012). At least 200 cells per sample were analyzed.

For lipid droplet visualisation in live cells, exponentially growing *S. pombe* cells were stained in suspension with 0.1 μg mL^−1^ BODIPY^TM^ 493/503 (Thermo Fisher Scientific) and briefly mixed by vortexing. No washes or sample dilution/concentration steps were performed to avoid stressing the cells or affecting their metabolism. Cells were centrifuged (1000 × g, 3 min, 25°C) and promptly imaged on soybean lectin-coated slides using the Olympus Cell R microscope. For imaging Ptl2-GFP, cells were fixed with 10% formaldehyde for 15 min, and washed three times with PBS, followed by microscopy. Fluorescent images were acquired as 16-bit Z-stacks (0.3 μm step size, 10 steps) in the green channel and were processed using the ImageJ software, version 1.51n (Schneider, Rasband and Eliceiri 2012) as maximum intensity projections. Care was taken to image all samples with the same exposure settings and to adjust brightness and contrast of all images identically in order to ensure adequate comparison of lipid droplet staining.

### Computational analysis of lipid droplet content

For every image, a mask corresponding to regions of dead or incompletely imaged cells was manually created. Each z-stack image was processed separately with a recursive thresholding method. The initial step was to separate cells from background using quantization to five levels. Further processing was done on the layer with the highest intensity by segmentation (Dougherty 2009). Dots with the lowest intensity were detected by this step. All segmented objects with area larger than 800 pixels or with non-circular shape were recursively taken to the next step of the segmentation process with a higher threshold value. The result of each level of the recursive thresholding method was a binary mask with segmented dots. The second step was to merge all segmented areas from the analyzed z-stacks. The last step was to remove detected objects that belonged to cells classified as dead or not present completely in the image, and objects having area smaller than 4 pixels. The final output of this process was a list of detected objects (i.e. lipid droplets) with extracted features. All processing was done in MATLAB Version: 9.2.0.556344 (R2017a) using Image Processing Toolbox Version 10.0 and Parallel Computing Toolbox Version 6.10.

## RESULTS

### The ‘cut’ phenotype of *Δcbf11* mutant manifests rapidly upon shift from EMM to YES medium

**Figure 1.**
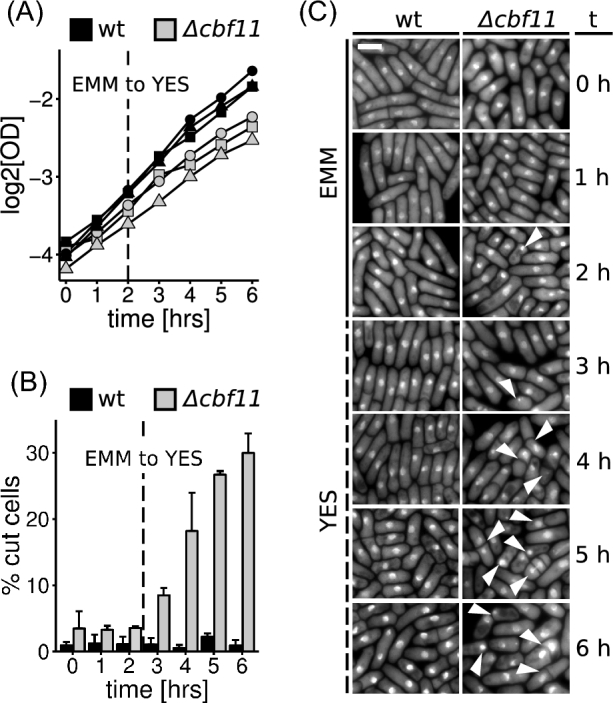
Dynamics of ‘cut’ phenotype occurrence in WT and *Δcbf11* upon shift from EMM to YES medium. (**A**) Growth curves of cells pre-cultured in EMM and then shifted to YES. No lag was apparent immediately after the shift. Data from three independent experiments are shown. (**B**) ‘cut’ *Δcbf11* cells start to accumulate within 1 hour after shift to YES. Mean values + SD of three independent experiments are shown. (**C**) Representative images of DAPI-stained cells from (B). ‘Cut’ cells are marked with arrowheads; scale bar = 5 μm.

We previously showed that *Δcbf11* cells grown as batch cultures in the minimal EMM medium showed improved growth rate and greatly diminished incidence of the ‘cut’ phenotype compared to *Δcbf11* cells grown in the complex YES medium (Převorovský et al. 2015, 2016). By performing a timecourse experiment, we now examined the dynamics of ‘cut’ occurrence upon shift from EMM to YES. Wild-type (WT) and *Δcbf11* cells were grown to exponential phase in EMM and culture aliquots were taken every hour. After 2 h of sampling, cells were collected by centrifugation, resuspended in YES and grown further. Cultures were sampled for four more hours and the frequency of ‘cut’ cells was determined by microscopy (Fig. 1). The medium shift did not show any immediate marked negative impact on culture growth rate, as determined by optical density measurements. However, at later timepoints the growth of *Δcbf11* cells slowed down significantly (timepoint 2 h vs 6 h; *P* = 0.0026; one-sided paired *t*-test). By contrast, the growth rate of WT cells did not decrease after the shift to YES (Fig. 1A). Strikingly, the occurrence of ‘cut’ phenotype in *Δcbf11* cells started to increase rapidly following the shift, reaching ∼30% at 6 h, whereas WT cells showed little change in ‘cut’ frequency (Fig. 1B, C). Given the slow growth rate of *Δcbf11* cultures in YES (Převorovský *et al.*^2009^), these results imply that already the very first mitosis after medium shift was affected in *Δcbf11* cells. Our previous results indicated that some nutrient(s) present in EMM but absent or limiting in YES are responsible for the observed mitotic defects, as opposed to a situation in which YES would contain a ‘poison’ component (Převorovský *et al.*^2015^). Such rapid response thus indicates that the beneficial nutrient(s) is depleted very fast upon medium shift and/or a rapid change in nutrient-dependent signaling is involved in triggering the ‘cut’ phenotype in the *Δcbf11* mutant.

### ‘Minerals’ component of EMM is responsible for growth rate suppression of *Δcbf11* cells

**Figure 2.**
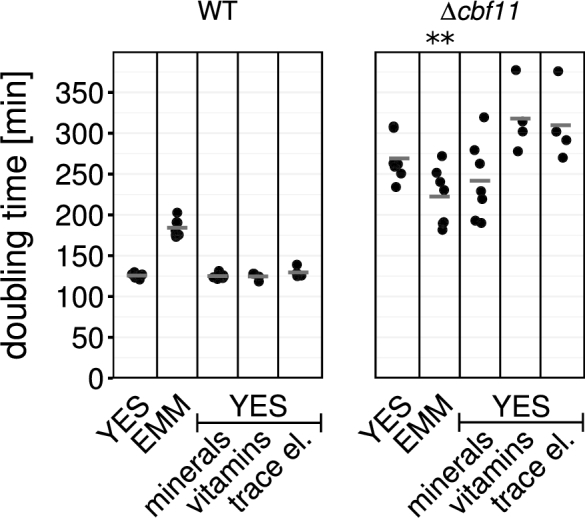
Effects of EMM medium components on doubling times of *Δcbf11* cultures. Addition of the ‘minerals’ group of EMM components can reduce the time required for biomass doubling of *Δcbf11* cells in YES. Data from ≥4 independent experiments are shown; horizontal lines represent mean values; significance of differences in *Δcbf11* doubling time in various media compared with *Δcbf11* grown in YES was tested by one-sided unpaired t-test: ** *P* ≤ 0.01.

We next wanted to identify the particular EMM component(s) responsible for suppressing defects in the *Δcbf11* mutant. We first focused on suppression of growth rate defects. We supplemented YES with various EMM components and compared growth with standard YES and EMM. According to the medium manufacturer, apart from glucose EMM is comprised of three categories of chemicals: ‘minerals’, ‘vitamins’ and ‘trace elements’ (see Table S1, Supporting Information). WT cells grow faster in YES than in EMM (Petersen and Russell 2016), and YES supplementation had little effect on their growth rate. Of the three groups of supplements, only ‘minerals’ could improve growth (i.e. reduce the doubling time) of *Δcbf11* cells when added to YES (Fig. 2), even though the growth improvement was not statistically significant. Nevertheless, any specific impact of ‘minerals’ on the mitotic defects of *Δcbf11* cells remained to be established.

### Addition of ammonium chloride to YES suppresses mitotic defects of *Δcbf11* and *cut-621* cells

**Figure 3.**
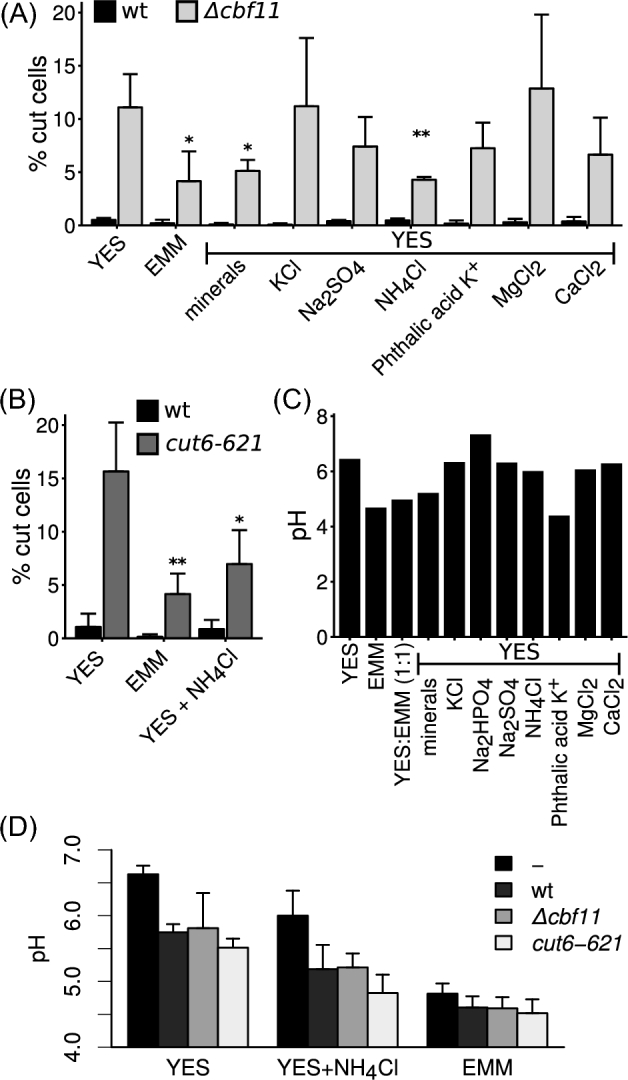
Effects of EMM medium components on ‘cut’ phenotype frequency in *Δcbf11* and *cut6–621* cultures. (**A**) Addition of the ‘minerals’ group of EMM components, and specifically addition of NH_4_Cl, suppress mitotic defects of *Δcbf11* cells in YES. (**B**) Addition of NH_4_Cl suppresses mitotic defects of *cut6–621* cells in YES at semi-permissive temperature. Mean values + SD of three independent experiments are shown in (A) and (B); significance of differences in ‘cut’ phenotype frequencies in *Δcbf11* or *cut6–621* in various media compared with corresponding mutant cultures grown in YES was tested by one-sided unpaired *t*-test: * *P* ≤ 0.05, ** *P* ≤ 0.01. (**C**) pH values for all media used in this study, before addition of cells. (**D**) pH values of selected conditioned media after overnight culture of the indicated strains. pH values of fresh media are indicated by „-‘; means + SD of three independent experiments are shown.

To directly test the impact of EMM components on ‘cut’ phenotype occurrence, we performed experiments with supplemented YES variants and analyzed the percentage of ‘cut’ cells by microscopy. We found that the ‘minerals’ component of EMM was able to suppress ‘cut’ incidence in *Δcbf11* cultures (Fig. 3A). To identify the specific component(s) responsible for this suppressive effect, we analyzed ‘cut’ phenotype frequencies in YES supplemented with individual components of the EMM ‘minerals’ mix (phthalic Acid K^+^, Na_2_HPO_4_, NH_4_Cl, MgCl_2_, CaCl_2_, KCl, Na_2_SO_4_). We observed strong ‘cut’ suppression in *Δcbf11* cells when ammonium chloride (NH_4_Cl), which serves as the nitrogen source in EMM, was added to YES (Fig. 3A). Both WT and *Δcbf11* cells failed to grow in YES supplemented with the ‘minerals’ component Na_2_HPO_4_ (data not shown).

Cbf11 is a regulator of gene expression with a pleiotropic mutant phenotype (Převorovský et al. 2009, 2015). To determine whether other, less pleiotropic, ‘cut’ mutants related to lipid metabolism can also be rescued by NH_4_Cl, we analyzed the *cut6–621* temperature-sensitive mutant at the semi-permissive temperature of 30°C. Expression of the *cut6* acetyl-coenzyme A carboxylase gene is regulated directly by Cbf11, and the *cut6–621* mutant also shows high frequency of the ‘cut’ phenotype when grown in YES at semi-permissive temperature (Převorovský *et al.*^2016^). Indeed, the *cut6–621* mitotic defects were diminished when NH_4_Cl was added to the growth medium (Fig. 3B), demonstrating that multiple lipid metabolism ‘cut’ mutants can be rescued by the addition of NH_4_Cl.

The Cut6 enzyme requires biotin for its function and biotin uptake is mediated by the Vht1 proton-biotin symporter, that functions optimally under acidic pH (Stolz 2003). Differences in the pH of the growth media might thus affect Cut6 function and ‘cut’ phenotype occurrence. Therefore, we measured the pH of the growth media used in this study (Fig. 3C). We found that while EMM was indeed more acidic than YES, there was little change in YES pH upon addition of NH_4_Cl. Furthermore, the only ‘minerals’ component able to lower pH when added to YES was potassium phthalate, which had no significant suppressive effect on ‘cut’ phenotype frequency (Fig. 3A). Since cellular metabolic processes might affect the cell's environment, we next measured pH of conditioned media in which cells had been grown overnight. As shown in Fig. 3D, the EMM medium is rather well buffered and its pH did not change much during cell cultivation. By contrast, both YES and YES supplemented with NH_4_Cl showed poor buffering capacity and became acidified during cell culture, but still remained more basic than both fresh and conditioned EMM. As shown in Fig. 1B, ‘cut’ *Δcbf11* cells appear rapidly after medium shift, so the factor affecting mitotic fidelity is likely present even in fresh, unconditioned media. Furthermore, we have previously shown that supplementing biotin in concentrations high enough to bypass the need for the Vht1 transporter had no suppressive effect on ‘cut’ frequency in *Δcbf11* cells (Stolz 2003; Převorovský *et al.*^2016^). It is therefore unlikely in this case that the pH of the medium plays a role in ‘cut’ phenotype suppression.

### Ammonium chloride boosts lipid droplet formation

Fatty acid synthesis and production of membrane phospholipids are required for mitotic nuclear envelope expansion and prevention of the ‘cut’ phenotype (Makarova *et al.*^2016^; Takemoto *et al.*^2016^). Since the addition of NH_4_Cl suppressed mitotic defects of lipid metabolism ‘cut’ mutants grown in YES (Fig. 3), we tested whether this suppression was associated with changes in lipid metabolism. To this end, we determined the distribution of lipid droplets in exponentially growing WT, *Δcbf11* and *cut6–621* cells cultured in YES (with or without NH_4_Cl) and EMM at 30°C. Lipid droplets are storage bodies composed of neutral triacylglycerols and sterol esters. Their formation shows cell-cycle and growth-phase dynamics (Long *et al.*^2012^; Meyers *et al.*^2017^) and they serve as a useful proxy for monitoring lipid biosynthesis (Rostron, Rolph and Lawrence 2015). Storage neutral lipids can be used to produce the membrane phospholipids required during mitosis (Makarova *et al.*^2016^). Interestingly, higher lipid droplet formation was found in *S. pombe* cells grown in EMM than in YES (He *et al.*^2014^).

**Figure 4.**
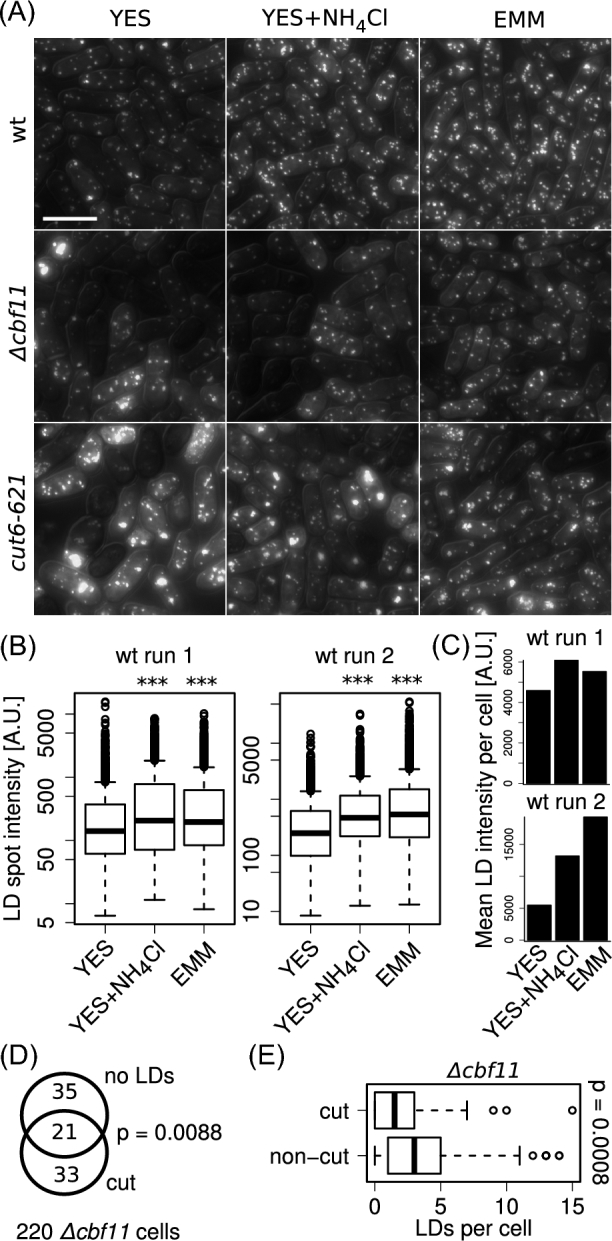
Lipid droplet formation in cells grown in YES, YES + NH_4_Cl, and EMM. (**A**) Representative images of WT, *Δcbf11* and *cut6–621* cells stained with BODIPY^TM^ 493/503 to visualize neutral lipids. DIC overlay is shown to mark cell boundaries. Scale bar = 10 μm. (**B**) Area-integrated staining intensities of individual lipid droplets in WT cells grown in the indicated media. Data from two independent experiments are shown; >180 cells were analyzed per sample. Significance of differences from distribution in YES was tested by one-sided unpaired Wilcoxon test: *** *P* ≤ 0.001. (**C**) Means of total lipid droplet signal intensities per WT cell. (**D**) *Δcbf11* cells with the ‘cut’ phenotype are enriched for cells lacking detectable lipid droplets (one-sided Fisher's exact test). (**E**) In the *Δcbf11* mutant, ‘cut’ cells contain fewer lipid droplets compared to ‘non-cut’ cells (one-sided unpaired Wilcoxon test).

First, we stained live, exponentially growing cells with BODIPY 493/503 to visualize neutral lipids. BODIPY dyes are insensitive to pH (Karolin *et al.*^1994^) and, thus, should not be affected in this regard by the different growth media used. Stained cells were then imaged using fluorescence microscopy, and lipid droplets were identified by computational image analysis. As shown in Fig. 4A-C, lipid droplets in WT cells grown in EMM or YES supplemented with NH_4_Cl were more abundant and typically had higher staining intensity than in cells grown in plain YES. This suggests that NH_4_Cl can indeed boost fatty acid and/or neutral lipid production in *S. pombe*. The *cut6–621* mutant grown at semi-permissive temperature in YES showed aberrant lipid droplet distribution with many cells having abnormally large, intensely stained lipid bodies, while some showing only very faint lipid droplet staining. According to our image analysis, this aberrant distribution was not significantly rescued in EMM or in YES with NH_4_Cl (data not shown). *Δcbf11* cells have decreased lipid droplet abundance when grown in YES and many cells do not contain any detectable lipid droplets at all. Both these phenotypes, low lipid droplet abundance and cell-to-cell heterogeneity, were largely rescued by growing *Δcbf11* cells in EMM (Fig. 4A and (Převorovský *et al.*^2015^)). By contrast, in YES with NH_4_Cl the population heterogeneity persisted, with some cells showing wild type-like lipid droplet patterns and others having very few or no detectable lipid droplets (Fig. 4A). No significant rescue in lipid droplet distribution by NH_4_Cl was detected by automated image analysis either (data not shown).

Next, we asked whether ‘cut’ cells specifically exerted any differences in lipid droplet content compared to ‘non-cut’ cells. We employed *Δcbf11* cells expressing GFP-tagged triacylglycerol lipase Ptl2, a marker of lipid droplets (Yang *et al.*^2016^). Cells growing exponentially in YES were fixed, stained with DAPI, and their lipid droplet content and nuclear phenotype were determined by microscopy and manual image analysis. We found a significant overlap between ‘cut’ cells and the subpopulation devoid of any detectable lipid droplets (Fig. 4D). Moreover, ‘cut’ *Δcbf11* cells had significantly lower numbers of lipid droplets compared to ‘non-cut’ *Δcbf11* cells (Fig. 4E), demonstrating association between the ‘cut’ phenotype and lipid droplet content in the *Δcbf11* mutant. The biological significance of this link and any causal relationships remain to be determined.

In summary, while ammonium chloride can boost lipid production in WT cells, it remains unclear whether it also boosts specifically the production of membrane phospholipids required for successful nuclear division. To clarify the issue, a more direct readout of membrane phospholipid synthesis in ‘cut’ mutants will be needed.

## DISCUSSION

Fission yeast mutants in the genes *cbf11* and *cut6* show the mitotic ‘cut’ phenotype when grown in complex medium (Saitoh *et al.*^1996^; Převorovský *et al.*^2009^). Cut6 is an essential acetyl-coenzyme A carboxylase involved in fatty acid synthesis, while Cbf11 is a transcription factor that regulates multiple genes involved in lipid metabolism, including *cut6*. We have previously shown that the mitotic defects in *Δcbf11* and *cut6–621* can be rescued by growing the cells in the minimal EMM medium (Převorovský et al. 2015, 2016). We have now identified ammonium chloride (NH_4_Cl), a major component of EMM and an efficiently utilized nitrogen source, as the compound specifically responsible for the rescue. Furthermore, we have shown that addition of NH_4_Cl increases lipid droplet content in WT cells, suggesting that NH_4_Cl affects fatty acid and/or neutral lipid metabolism.

The YES medium is often referred to as ‘rich’, which is rather confusing in terms of nitrogen content. Based on information from various media manufacturers, YES typically contains ∼0.06% nitrogen, embedded in a range of substances such as amino acids, nucleobases and vitamins. These substances differ in the ease with which their nitrogen is utilized by fission yeast cells, many of them representing ‘poor’ nitrogen sources (Petersen and Russell 2016). Moreover, a complex enzymatic apparatus is required to utilize such varied mixture of nitrogen sources. On the other hand, EMM contains ∼0.13% nitrogen, more than twice as much as YES does. Importantly, the vast majority of this nitrogen is present as just one substance—ammonium chloride, a ‘good’ nitrogen source. Thus, with regard to nitrogen, EMM is the ‘rich’ medium, not YES. Since *S. pombe* shows exquisite sensitivity to both nitrogen source quantity and quality (Davie, Forte and Petersen 2015), the difference between YES and EMM is significant and concordant with the observed differential effects of these media on cell physiology and mitotic fidelity.

We found that NH_4_Cl boosted lipid droplet content in exponentially growing WT cells (Fig. 4A). Curiously, accumulation of lipids and/or lipid droplets in yeasts has previously been linked rather with nutrient-poor conditions, such as stationary phase in *S. pombe* (Meyers *et al.*^2017^), nitrogen limitation in *Yarrowia lipolytica* (Kerkhoven *et al.*^2016^), or TORC1 inhibition by rapamycin in *S. cerevisiae* (Madeira *et al.*^2015^). However, increased lipid droplet content in EMM vs YES has already been reported by others for *S. pombe* (He *et al.*^2014^). Importantly, the lipid droplet increase triggered by NH_4_Cl or EMM is modest (i.e. increased ‘baseline exponential content’) compared to the pronounced lipid droplet accumulation in stationary *S. pombe* cells. Therefore, these might be two independent phenomena with distinct regulation, each taking place at a different stage of culture growth.

The ‘cut’ phenotype occurrence upon perturbation of lipid metabolism has been previously ascribed to insufficient fatty acid production and membrane phospholipid supply during anaphase, when rapid expansion of the nuclear envelope takes place. Hampered nuclear elongation during the anaphase of a closed mitosis then results in the collapse of the division spindle (Makarova *et al.*^2016^; Takemoto *et al.*^2016^). So what could be the mechanism whereby NH_4_Cl rescues the ‘cut’ mitotic defects? One possibility is that NH_4_Cl increases membrane phospholipid production, thereby providing mutants with compromised lipid metabolism with the required nuclear envelope components. While we found association between the ‘cut’ phenotype and decreased lipid droplet content in the *Δcbf11* strain, we did not see any significant corrective effect of NH_4_Cl on lipid droplet amount and distribution in either *Δcbf11* or *cut6–621* mutant cells (Fig. 4). Since lipid droplet content represents a complex and only indirect readout of phospholipid production capacity and/or might not be reliable in mutants with deregulated lipid metabolism, the nature of the NH_4_Cl-mediated suppression of mitotic defects with regard to lipid metabolism needs to be studied further, using more direct readouts. Interestingly, lipid metabolism is extensively regulated during the cell cycle in both yeast and human cells (the latter featuring an open mitosis) and this dynamics is important for successful cell division (Stumpf *et al.*^2013^; Atilla-Gokcumen *et al.*^2014^; Blank *et al.*^2017^).

NH_4_Cl is not directly utilized in fatty acid synthesis, so any NH_4_Cl-dependent changes in lipid metabolism are likely brought about indirectly, possibly via nitrogen-sensitive signaling pathways. Indeed, the mitotic defects of *Δcbf11* cells are also rescued by mutations in major nutrient-responsive kinases, *pka1* and *sty1* (Převorovský *et al.*^2015^), both of which are sensitive to nitrogen availability (Maeda *et al.*^1994^; Shiozaki and Russell 1996). Moreover, the lethality of the temperature-sensitive ‘cut’ mutants in *cut4* and *cut9*, encoding subunits of APC/C likely unrelated to lipid metabolism, can be rescued by PKA inactivation and/or by growing the cells in EMM. Nevertheless, the specific impact on ‘cut’ occurrence or the particular nutrient(s) responsible for the suppression were not analyzed (Yamashita *et al.*^1996^; Yamada, Kumada and Yanagida 1997). Therefore, a common theme emerges where ‘cut’ phenotype caused by a broad range of defects can be rescued by altered nutrient availability and/or sensing. However, it remains to be established whether there is one common molecular mechanism of nutrient-related ‘cut’ suppression, or whether multiple (complementary) mechanisms exist. One possible speculative mechanism involves NH_4_Cl acting via Pka1/Sty1-dependent signaling to modulate lipid metabolism in a manner promoting production of membrane phospholipids, thus supporting timely nuclear envelope expansion and faithful mitotic progression.

In future, it will be interesting to determine how fission yeast cells (and particularly ‘cut’ mutants related to lipid metabolism) respond to different doses of NH_4_Cl. It will also be important to examine the effects of other ammonium sources (e.g. ammonium sulfate, which is used in some fission yeast media) and other nitrogen sources, in general, including suboptimal ones. The potential involvement of TOR signaling, another major nitrogen-responsive regulator, in the suppression of the ‘cut’ phenotype should also be addressed.

## Supplementary Material

Supplement Table 1
